# Data on the effects of losartan on protein expression, vascular reactivity and antioxidant capacity in the aorta of ethanol-treated rats

**DOI:** 10.1016/j.dib.2017.01.006

**Published:** 2017-01-17

**Authors:** Carla S. Ceron, Gabriel T. do Vale, Janaina A. Simplicio, Patrícia Passaglia, Sthefany T. Ricci, Carlos R. Tirapelli

**Affiliations:** a Laboratório de Farmacologia, DEPCH, Escola de Enfermagem de Ribeirão Preto, USP, Ribeirão Preto, SP, Brazil; b Departamento de Farmacologia, Faculdade de Medicina de Ribeirão Preto, Universidade de São Paulo (USP), Ribeirão Preto, SP, Brazil

**Keywords:** Ethanol, Losartan, Oxidative stress, Reactive oxygen species, Vascular dysfunction

## Abstract

We describe the effects of losartan, a selective AT_1_ receptor antagonist on the alterations induced by treatment with ethanol in the rat aorta. The data shown here are related to the article entitled “Angiotensin type 1 receptor mediates chronic ethanol consumption-induced hypertension and vascular oxidative stress” (P. Passaglia, C.S. Ceron, A.S. Mecawi, J. Antunes-Rodrigues, E.B. Coelho, C.R. Tirapelli, 2015) [1]. Here we include new data on the protective effect of losartan against ethanol-induced oxidative stress. Male Wistar rats treated for 2 weeks with ethanol (20%, vol./vol.) exhibited increased aortic production of reactive oxygen species (ROS) and losartan (10 mg/kg/day; p.o. gavage) prevented this response. Ethanol did not alter the expression of eNOS in the rat aorta. Losartan prevented ethanol-induced increase in the aortic expression of nNOS. Neither ethanol nor losartan affected superoxide dismutase (SOD) or catalase (CAT) activities in the rat aorta. Treatment with ethanol increased the contraction induced by phenylephrine in both endothelium-intact and endothelium-denuded aortas and these responses were prevented by losartan. Conversely, neither ethanol nor losartan affected the endothelium-dependent relaxation induced by acetylcholine.

**Specifications Table**

**Table:** 

Subject area	Biology
More specific subject area	Vascular Pharmacology
Type of data	Graph, image
How data was acquired	Microscope (Leica Model SPE, Leica Imaging Systems Ltd., Wetzlar, Germany), chemiluminescence (Orion II luminometer, Berthold Detection Systems, Pforzheim, Germany), spectroscopy, colorimetric tests, isometric force transducer (TRI201; Panlab, Barcelona, Spain)
Data format	Analyzed
Experimental factors	Male Wistar rats initially weighting 250–280 g were treated with increasing doses of ethanol (solutions of 5 and 10% in the 1st and 2nd week and 20% from the 3rd to the 5th week). Some rats were orally treated with losartan (10 mg/kg/day, gavage).
Experimental features	ROS generation was evaluated by fluorescence using dihydroethidium (DHE) and lucigenin chemiluminescence. Protein expression was determined by western immunoblotting. SOD and CAT activities were determined colorimetrically and spectrophotometrically, respectively. Vascular reactivity experiments were performed in organ baths using an isometric force transducer.
Data source location	Ribeirao Preto, Brazil
Data accessibility	Data are within this article

**Value of the data**

• Ethanol-induced protein expression and vascular dysfunction are dependent on AT_1_ receptor activation.
• Losartan can prevent ethanol-induced ROS generation in the vasculature.
• The data provide insights into the mechanisms whereby ethanol affects the vasculature.

## 1 Data

**Fig. 1:**
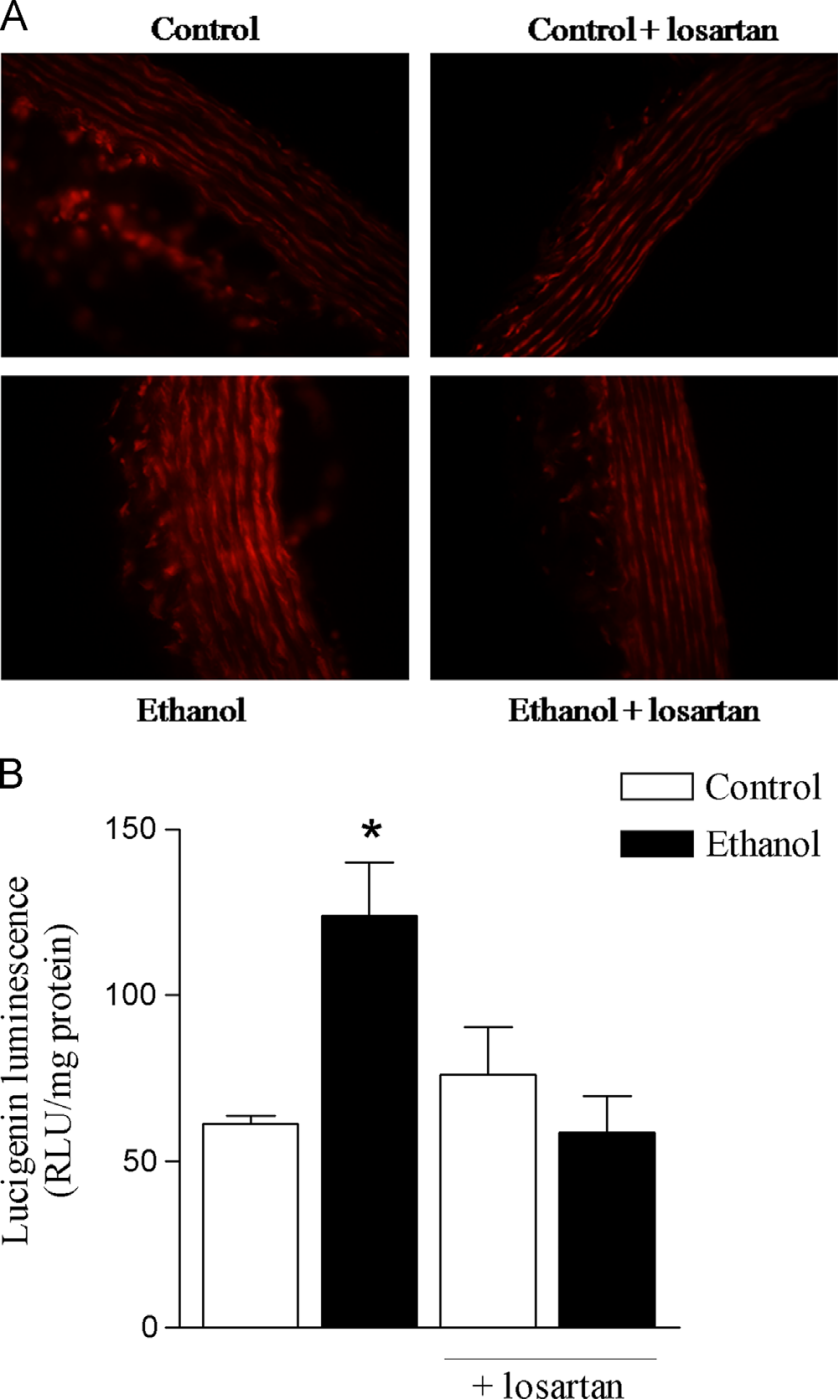
Protective effects of losartan against ethanol-induced ROS generation in the rat aorta. (A) ROS visualization in aorta slices using the fluorescent dye dihydroethidium (DHE). (B) Generation of superoxide anion quantified by lucigenin-derived luminescence. Results are shown as mean±SEM of 6–8 experiments. *Compared to control, control+losartan and ethanol+losartan (*p*<0.05, ANOVA followed by Newman–Keuls).

**Fig. 2:**
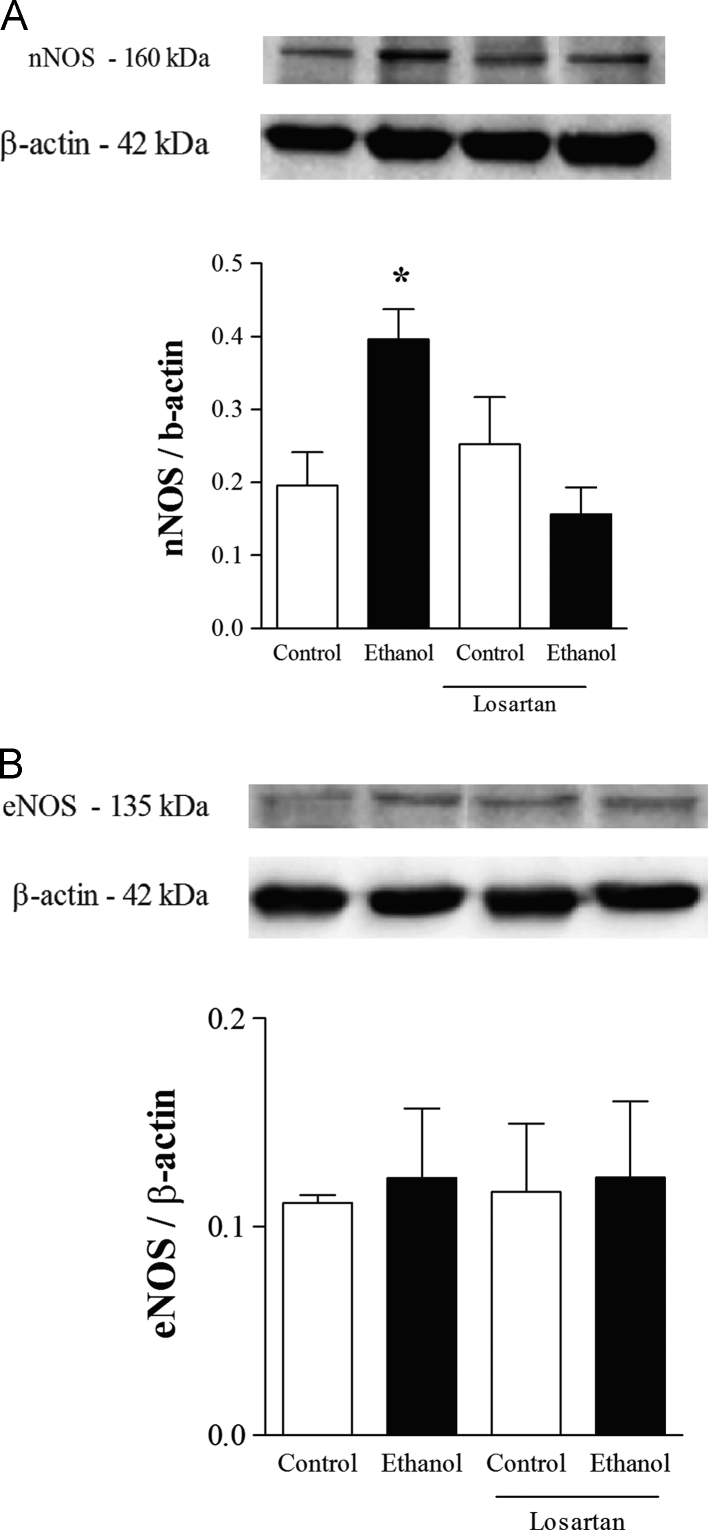
Expression of nNOS and eNOS in aortas from ethanol-treated rats. Top panels: representative immunoblots for NOS isoforms. Bottom panels: corresponding bar graphs show densitometric data for protein expression of (A) nNOS and (B) eNOS. Results are shown as mean±SEM of 4–5 experiments for each group. *Compared to control, control+losartan and ethanol+losartan (*p*<0.05, ANOVA followed by Newman–Keuls).

**Fig. 3:**
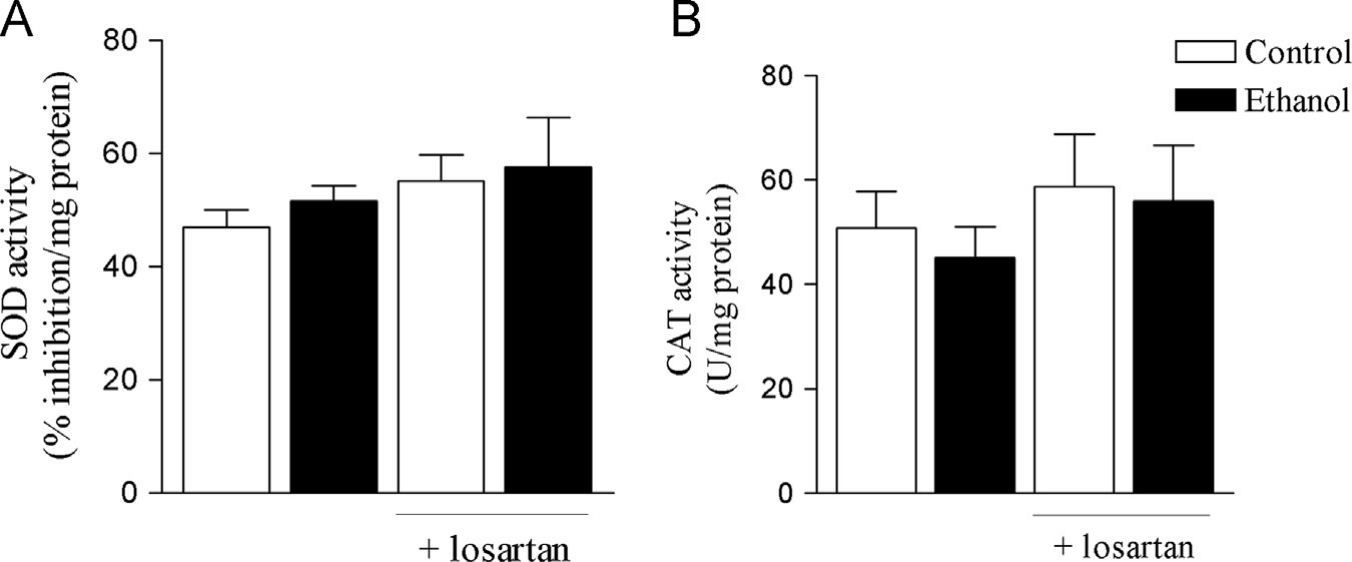
SOD and CAT activities in aortas from ethanol-treated rats. (A) SOD and (B) CAT activities were determined in the rat aorta. Results are shown as mean±SEM of 5 to 8 animals.

**Fig. 4:**
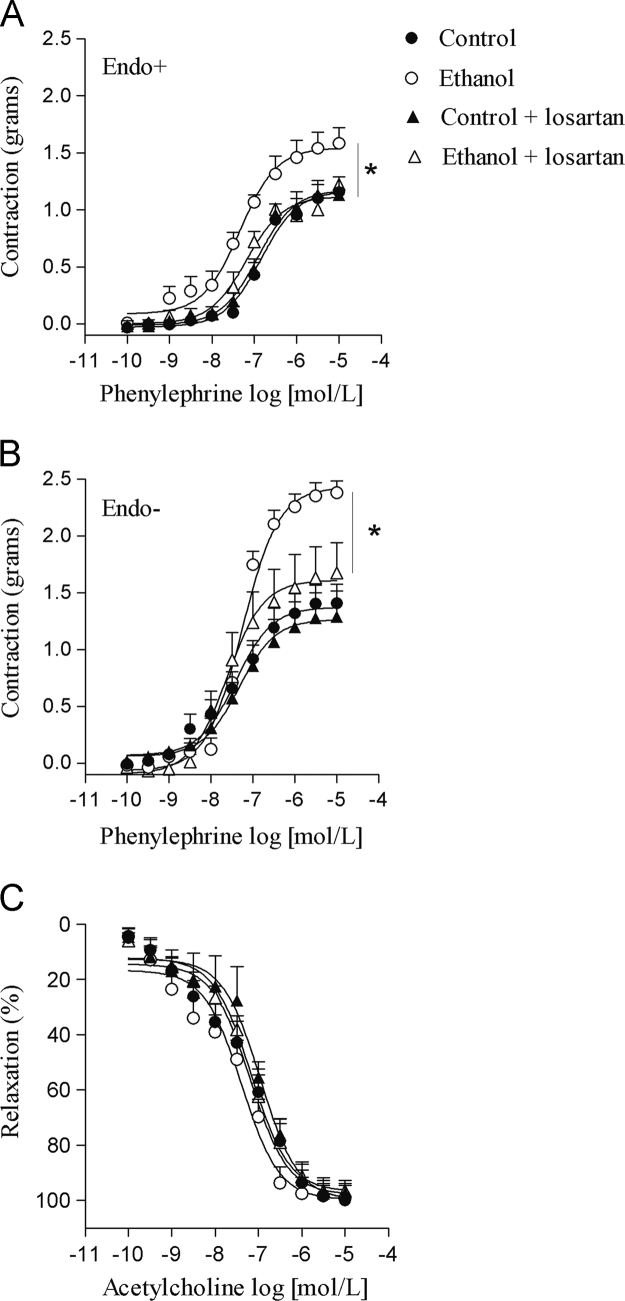
Protective effects of losartan against ethanol-induced vascular dysfunction. (A) Concentration–response curves for phenylephrine in endothelium-intact (Endo+) aortas; (B) Concentration–response curves for phenylephrine in endothelium-denuded (Endo−) aortas; (C) Concentration–response curves for acetylcholine in endothelium-intact aortas. Values are mean±SEM of 7–9 independent preparations. *Compared to control, control+losartan and ethanol+losartan (*p*<0.05, ANOVA followed by Newman–Keuls).

Data describe the protective effects of losartan against ethanol-induced vascular dysfunction. Losartan prevented the increase in ROS generation induced by ethanol in the rat aorta (Fig. 1). The increase in the aortic expression of nNOS induced by ethanol was prevented by losartan as shown on Fig. 2. Neither losartan nor ethanol affected SOD or CAT activities in the rat aorta (Fig. 3). Treatment with ethanol increased phenylephrine-induced contraction in both endothelium-intact and endothelium-denuded aortas and losartan prevented these responses (Fig. 4A and B). Acetylcholine-induced relaxation was not affected by ethanol or losartan (Fig. 4C). No differences on pD_2_ values were detected among groups (data not shown).

## 2 Experimental design, materials and methods

Male Wistar rats were anaesthetized with urethane 1.25 g/kg (i.p.) and the thoracic aorta was cleaned and isolated.

### 2.1 Evaluation of oxidative stress and protein expression

DHE images were obtained as previously described [2]. The assays carried out to evaluate lucigenin-derived luminescence, protein expression and SOD or CAT activities were performed as described elsewhere [1], [2].

### 2.2 Vascular reactivity studies

Vascular reactivity experiments were carried out as previously described [2], [3]. Concentration–response curves for phenylephrine or acetylcholine were analyzed by non-linear regression fit and the maximal effect (Emax) elicited by phenylephrine is expressed on grams, whereas Emax values for acetylcholine are expressed as % relaxation from pre-contractile levels induced by phenylephrine. The potency of both drugs was calculated and expressed as pD_2_ values, which corresponds to the negative logarithm of the EC_50_ values [3].

### 2.3 Statistical analysis

Results are expressed as means ± standard error of the mean (SEM). Two-way analysis of variance (ANOVA) followed by Newman–Keuls multiple comparison test were used to analyze the data. *P* values of less than 0.05 were considered significant.
